# Stepping strong: Nurse-led support for mothers in clubfoot care

**DOI:** 10.6026/973206300213682

**Published:** 2025-10-31

**Authors:** Kavitha Ethiraj, Shankar Shanmugam Rajendran, Arun Mozhi Rajan, Kannan Kasinathan, Grace Anita Samuel, Jency Indira kuppan, Chitra Gopalan

**Affiliations:** 1 Department of Child Health Nursing, College of Nursing, Madras Medical College, The Tamil Nadu Dr. M.G.R. Medical University, Chennai, Tamil Nadu, India; 2 Department of Paediatric Orthopaedics, College of Nursing, Madras Medical College, The Tamil Nadu Dr. M.G.R. Medical University, Chennai, Tamil Nadu, India; 3 Department of Obstetrics and Gynaecological Nursing, College of Nursing, Madras Medical College, The Tamil Nadu Dr. M.G.R. Medical University, Chennai, Tamil Nadu, India; 4 Department of Medical And Surgical Nursing, College of Nursing, Madras Medical College, The Tamil Nadu Dr. M.G.R. Medical University, Chennai, Tamil Nadu, India

**Keywords:** Clubfoot, nurse-led care programme, self-efficacy, quality of life, caregiver burden

## Abstract

The effectiveness of a nurse-led intervention in enhancing self-efficacy and quality of life (QoL) among mothers of children with clubfoot is of interest. Sixty mothers were selected and received structured educational sessions on child care. Data shows significant improvements in maternal self-efficacy and QoL, with all QoL domains showing statistically significant gains. The study highlights the importance of nurse-led interventions in enhancing maternal caregiving capabilities. Thus, we show that such programs contribute to better treatment adherence and overall well-being for mothers.

## Background:

Clubfoot, also known as congenital talipes equinovarus (CTEV), is a common congenital musculoskeletal deformity that affects 1 to 2 per 1,000 live births globally, with a higher prevalence in low- and middle-income countries [1]. If left untreated or poorly managed, it leads to permanent impairment, gait abnormalities and significant emotional challenges for both the child and caregiver. Early detection and standardized treatments, such as the Ponseti method, have significantly improved outcomes, with success rates exceeding 90% [2]. However, the treatment process often spans several years, requiring constant caregiver involvement, including casting, brace management and regular hospital visits [3]. This extended care imposes considerable emotional, financial and physical strain, particularly on mothers who are typically the primary caregivers [4]. Self-efficacy, as defined by psychologist Albert Bandura, refers to an individual's belief in their ability to perform tasks necessary to achieve desired outcomes [5]. In the context of clubfoot care, high self-efficacy in mothers leads to greater confidence in managing medical routines, problem-solving and decision-making, which directly impacts treatment adherence and stress levels [6]. Conversely, low self-efficacy can hinder treatment compliance and negatively affect both the caregiver's and child's well-being. Enhancing self-efficacy among caregivers is, therefore, a critical strategy for improving health outcomes for children with clubfoot [7]. Quality of life (QoL) for caregivers, especially mothers, is often compromised due to the physical and emotional demands of caregiving, societal stigma and stress. Studies show that psychological and environmental domains of QoL are most affected, underscoring the need for supportive interventions. Improving QoL not only benefits the caregiver but also positively influences the child's treatment adherence and overall development [8]. Nurse-led interventions, including educational programs, have proven to be a practical approach to managing chronic conditions [9]. These programs aim to empower caregivers by enhancing their knowledge and self-efficacy, thereby improving their overall psychosocial well-being [10]. This study aims to evaluate the impact of nurse-led care programs on improving self-efficacy and QoL in mothers of children with clubfoot, providing evidence for integrated, family-centered care in pediatric orthopedics. Therefore, it is of interest to determine the effectiveness of nurse-led care programs in enhancing self-efficacy and quality of life among mothers of children with clubfoot, ultimately supporting better health outcomes and integrated care in pediatric orthopedics.

## Materials and Methods:

A quantitative research methodology was utilised to evaluate the effect of a nurse-led care program on self-efficacy and quality of life (QoL) in mothers caring for children with clubfoot. The study utilised a pre-experimental one-group pre-test post-test design, enabling the researcher to compare pre- and post-intervention results. The research was conducted at the Orthopaedic Department of the Institute of Child Health and Hospital for Children, Egmore, Chennai - 600 008, over a period of four weeks. A non-probability convenience sampling method was employed to select 60 mothers who met the inclusion criteria: mothers aged 19-45 years, capable of reading Tamil or English and caring for children with unilateral or bilateral clubfoot. Mothers of children with other congenital defects, those with severely ill children and those engaged in different research studies were eliminated. The data collection instruments comprised a demographic proforma, the Caregiver Self-Efficacy in Contribution to Self-Care Scale and the WHOQOL-BREF Scale. The nurse-led intervention comprised a structured 30-40 minute session that included training in care of a child with casting, brace application, home exercise techniques and complication management to enhance self-efficacy, as well as counselling on nutrition, sleep, play activities, social support systems and peer bonding to improve quality of life, supplemented by educational booklets. A post-test evaluation utilising the same scales was performed 21 days after the intervention. Ethical approval was secured from the Institutional Ethics Committee of Madras Medical College, Chennai-03 (IEC-MMC/Approval/42112024). Informed consent was obtained from every participant and the study rigorously adhered to ethical standards, encompassing autonomy, beneficence, confidentiality and justice. The data were analysed utilising SPSS version 22. A significance level of p < 0.05 was deemed statistically significant.

## Results:

The study evaluated the effectiveness of nurse-led care interventions on self-efficacy and quality of life, with the results summarized in Tables 1 (see PDF) and 2 (see PDF). Table 1 (see PDF) presents the effectiveness of the nurse-led care intervention on self-efficacy. It shows that, prior to the intervention, 78.33% of participants had low self-efficacy, while only 21.67% had moderate self-efficacy, and no participants exhibited high self-efficacy. After the intervention, 71.67% of participant's demonstrated high self-efficacy, 28.33% had moderate self-efficacy, and none had low self-efficacy. The Chi-square test revealed a highly significant improvement in self-efficacy, with a p-value of 0.001, indicating that the intervention had a substantial effect on increasing self-efficacy levels among the participants. Table 2 (see PDF) outlines the impact of the nurse-led care intervention on quality of life. Initially, 75% of participants reported a low quality of life, 25% had a moderate quality of life and none had a high quality of life. After the intervention, 60% of participants reported a moderate quality of life, and 40% reported a high quality of life. The Chi-square test indicated a highly significant improvement in quality of life, with a p-value of 0.001, demonstrating that the intervention effectively enhanced the participants' quality of life.

Correlation analysis revealed a moderate positive relationship between self-efficacy and quality of life following the nurse-led intervention (r = 0.43, p = 0.01). This indicates that as mothers' confidence and perceived ability to manage their child's condition increased, their quality of life also improved. Post-test self-efficacy showed significant associations with mother's age (χ^2^=7.35, p=0.05), educational qualification (χ^2^=9.50, p=0.05) and residential status (χ^2^=7.12, p=0.05). Mothers aged over 30 years and those with higher academic qualifications demonstrated higher self-efficacy, indicating that age-related maturity and educational exposure enhanced their caregiving capacity. Urban residents also demonstrated higher self-efficacy than their rural or suburban counterparts, likely reflecting better access to healthcare. Quality of life (QOL) was significantly associated with family income (χ^2^ = 5.99, p = 0.05), residence (χ^2^ = 6.66, p = 0.05) and birth order (χ^2^ = 6.55, p = 0.05). Higher-income urban families and mothers of third-born children had better QOL, possibly due to financial stability and greater parenting experience. Other variables were not significant.

## Discussion:

The present study demonstrated that a nurse-led care programme significantly enhanced maternal self-efficacy and quality of life (QOL) among mothers of children with clubfoot. Initially, most mothers had low self-efficacy (78.33%) and QOL (75%), with none achieving high scores. After the intervention, 71.67% achieved high self-efficacy and 28.33% moderate self-efficacy, with a notable mean score improvement of 16.34 points (t = 46.27, p = 0.001). These results are consistent with those of Mohammed *et al.* (2025) [11] and Agarwal *et al.* (2025) [12], who found that nurse-led intervention programs significantly improved mothers' knowledge of effective parenting and caregivers' self-efficacy, especially in psychological and environmental domains (p < 0.001), which improved with counseling and support. While Agarwal et al. focused on QOL stabilization, this study extends the evidence by demonstrating significant gains in maternal self-efficacy, a key factor for treatment adherence and overall well-being. Post-test results showed that self-efficacy was significantly associated with mothers' age, education and residence, with higher scores among older, educated, urban mothers. Quality of life correlated with income, residence and birth order, indicating better outcomes among higher-income urban families and mothers with more parenting experience. Other demographic factors were not significant. The findings align with those of Dong *et al.*, indicating that demographic characteristics, including maternal age, education and income, influence caregiving capacity and psychological resilience. Older, educated mothers in urban areas reported higher self-efficacy and quality of life, similar to the improvements in psychological stress observed with Ponseti treatment. This suggests socioeconomic stability and effective interventions reduce maternal stress and enhance caregiving outcomes.

## Conclusion:

We show that nurse-led care programs significantly improved maternal self-efficacy and quality of life among mothers caring for children with clubfoot. Post-intervention gains were evident across all QOL domains, with demographic factors such as age, education, income and residence influencing outcomes. Thus, we show the importance of integrating structured nurse-led interventions into clubfoot care to enhance caregiver capacity, reduce psychosocial burden and promote treatment adherence. Addressing both clinical and psychosocial dimensions is crucial for achieving comprehensive outcomes in the management of congenital musculoskeletal deformities.
